# Multi-Temperature Crystallography of S-Adenosylmethionine Decarboxylase Observes Dynamic Loop Motions

**DOI:** 10.3390/biom15091274

**Published:** 2025-09-03

**Authors:** Jenitha R. Patel, Timothy J. Bonzon, Timothy F. Bakht, Omowumi O. Fagbohun, Jonathan A. Clinger

**Affiliations:** Department of Chemistry and Biochemistry, Baylor University, One Bear Place 97348, Waco, TX 76798, USA; jenitha_patel1@baylor.edu (J.R.P.); timothy_bonzon1@baylor.edu (T.J.B.); timothy_bakht1@baylor.edu (T.F.B.); omowumi_fagbohun1@baylor.edu (O.O.F.)

**Keywords:** S-adenosylmethionine decarboxylase, polyamine, protein dynamics, protein crystallography

## Abstract

S-adenosylmethionine decarboxylase (AdoMetDC) is an essential enzyme in the polyamine biosynthesis pathway and plays a key role in the synthesis of the polyamines spermidine and spermine, polycationic alkylamines that are present in millimolar levels in mammalian cells. Polyamines are metabolic molecules that are involved in many fundamental processes, including regulation of protein and nucleic acid synthesis, stabilization of chromatin, differentiation, apoptosis, protection from oxidation, and regulation of ion channels. Multiple oncogenic pathways lead to dysregulation of polyamines, making polyamines a potential biomarker for cancer and polyamine biosynthesis a target for therapeutic intervention. This study uses multi-temperature crystallography to probe the structure and dynamics of AdoMetDC by collecting diffraction data at 100 K, 273 K, and 293 K. Differential loop behavior is observed across the collected datasets, with dramatic residue rearrangements. In the loop containing residues 20–28, the ambient temperature datasets show a large motion relative to the cryo structure. In a second loop containing residues 164–174, previous cryo structures do not report ordered positions. This loop is ordered in our 100 K structure, while assuming different conformations in the 273 K and 293 K data. These results further illustrate the usefulness of ambient data collection for understanding the structure and dynamics of proteins, especially in loop regions which are less restrained than protein cores.

## 1. Introduction

**Figure 1:**
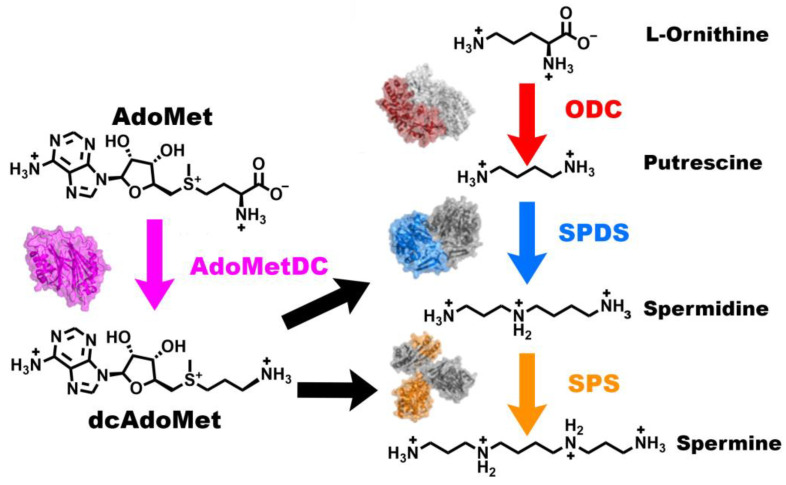
Polyamine biosynthesis pathway with structures of human enzymes. In the co-first steps, S-adenosylmethionine (AdoMet) and L-ornithine are converted to decarboxylated S-adenosylmethionine (dcAdoMet) and putrescine by AdoMetDC (magenta PDB ID 1JEN) and ODC (red/gray PDB ID 1D7K), respectively. Putrescine is then converted to spermidine using dcAdoMet as the aminopropyl donor by SPDS (blue/gray PDB ID 2O07). Spermidine is then converted to spermine using a second dcAdoMet as an aminopropyl donor by SPS (orange/gray PDB ID 3C6K). Gray structures indicate the second chain of homodimer pairs.

Polyamines are polycations that are found in life across phyla and are important for many cellular growth processes [1,2]. All eukaryotes can synthesize their own polyamines via the polyamine synthesis pathway, and polyamines are essential for cell growth and proliferation in eukaryotes [3]. Balancing polyamine levels is essential, as deviations from their narrow ideal range has severe physiological consequences [4,5]. In humans, the polyamine biosynthetic pathway enzymes consist of ornithine decarboxylase (ODC), S-adenosylmethionine decarboxylase (AdoMetDC), spermidine synthase (SPDS), and spermine synthase (SPS) [6,7]. AdoMetDC converts S-adenosylmethionine (AdoMet) to decarboxylated S-adenosylmethionine (dcAdoMet). dcAdoMet is then used as the aminopropyl donor for SPDS and SPS for their reactions with putrescine and spermidine, respectively [8,9,10] (Figure 1).

Due to the important functions of the polyamine biosynthesis pathway and its association with various disease states, the pathway has long been a target for development of therapeutics. AdoMetDC specifically is rate-limiting for the formation of spermidine and spermine, making it an attractive therapeutic target for modulating polyamine synthesis and cellular levels. A number of inhibitors and drug candidates for AdoMetDC have been proposed and tested in clinical trials [11,12,13,14]. The search for new and improved inhibitors of AdoMetDC and other enzymes in the polyamine biosynthesis pathway is on-going, including recent efforts to use virtual screening as well as development of irreversible inhibitors [15,16,17].

AdoMetDC is a decarboxylase which depends on a pyruvoyl cofactor for its activity, unlike the more common pyridoxal-5′-phosphate dependent enzymes [18]. Other examples of the pyruvoyl-dependent decarboxylation enzymes include aspartate decarboxylase, histidine decarboxylase, and arginine decarboxylase [19,20,21,22,23]. AdoMetDC is expressed as a proenzyme and auto-processes into the active form by an internal serinolysis reaction leading to backbone cleavage to *α* and *β* subunits and creation of the pyruvoyl group at the N-terminus of the larger *α* subunit [24]. This process is activated in humans in the presence of putrescine. Human AdoMetDC (hAdoMetDC) is a dimer in solution according to analytical ultracentrifugation with a 3 × 10^7^ M^−1^ dimerization constant and has been found to cooperatively bind the activating putrescine [25]. However, AdoMetDC concentration is tightly controlled in the living cell by multiple mechanisms, including translational repression, and the cellular half-life of AdoMetDC is as little as one hour [6,26,27]. Previous structural and mutagenic work of this enzyme has been vital to understanding the activation, selectivity, and mechanistic behavior of this enzyme [24,25,28,29,30,31].

Even though many structural studies have provided a deep wealth of knowledge about this enzyme, there are still some gaps in our structural understanding of this enzyme. Previously solved crystal structures contain multiple unmodeled flexible loops, particularly loops containing residues 20–28 (disordered loop 1, DL1), 164–174 (disordered loop 2, DL2), and 292–302 (disordered loop 3, DL3). DL1 and DL2 form a pocket/cleft across from the AdoMet binding pocket. Previous data collections have demonstrated that DL1 becomes more ordered in structures that contain AdoMet mimics (3DZ2, 3DZ3, and 3DZ5) or inhibitors (1I7C, 1I7B, and 1I7M). DL2 has also been reported to be more ordered in a subset of structures containing AdoMet mimics and inhibitors (Figure S1). Most interestingly, a single ambient dataset has been previously collected, containing the methylglyoxal bis(guanylhydrazone) (MGBG) inhibitor [31]. MGBG was the first discovered inhibitor of AdoMetDC, and it binds tightly to the entrance to the AdoMetDC active site [11,31]. This dataset, while lower resolution (2.49 Å), is the only structure to fully model DL2. It also reports more structure in DL1 than many other AdoMetDC depositions, only leaving residues 24–26 unmodeled. Other inhibitor bound states, such as the structure containing the AdoMet mimic 5′-[(3-aminopropyl)methylamino]-5′deoxy-8-methyladenosine (PDB ID 3DZ2), leave 23–26 unmodeled [30]. They also typically leave smaller gaps than the apo structure in DL2, but gaps between residues 166–171 are typical versus the 164–174 gap in apo [29,30]. The variable amounts of order in the datasets generally correspond to more order in structures that contain active site ligands which indicates that DL1 and DL2 are at minimum sensitive to active site perturbation, even though the mechanism of their ordering remains unclear. If the active site structure can perturb the structure of these loops, they could also possibly drive active site conformations as well, which may have implications for inhibitor design by targeting these pockets.

To probe the influence of temperature on the AdoMetDC structure, as the MCBG structure suggests may be possible, we performed multi-temperature crystallography experiments on the apo form of AdoMetDC. Structures previously collected above the glass transition and closer to the physiological temperature in other systems demonstrate structural transitions associated with data collection temperature [32,33,34,35,36,37,38,39,40,41,42,43,44,45,46]. Previous reported results from multi-temperature work in other systems include allosteric loop opening and closing, as well as alternative ligand binding poses [34,35,38,40,41]. In this study, crystallographic data was collected at 273 K and 293 K as well as 100 K to probe for differences in the structures according to data collection temperature. The new structures of human AdoMetDC reported here have altered loops as a function of data collection temperature, indicating that this enzyme’s structure is affected by cryo-cooling and/or cryoprotection. The new structures improve our understanding of the structural ensemble and conformations that are present at closer to physiological temperatures.

## 2. Materials and Methods

### 2.1. Protein Expression and Purification

The human S-adenosylmethionine decarboxylase gene *AMD1* was codon-optimized for *E. coli* expression and synthesized (Genscript, Piscataway, NJ, USA). It was cloned into a pET27b vector with an N-terminal 6 × HisTag upstream of a tobacco etch virus protease cleavage site. This vector was transformed into BL21 cells for hAdoMetDC expression. For large-scale expression, the cells were introduced to modified terrific broth media (12 g/L tryptone, 24 g/L yeast extract, 0.4% *v*/*v* glycerol, and 0.017 M potassium phosphate monobasic (all Fisher Scientific, Hampton, NH, USA)), containing 50 μg/mL kanamycin from overnight starter cultures. Cultures were shaken at 37 °C and 225 rpm until optical density of 0.6 was reached. Then, 0.5 mM isopropyl *β*-D-1-thiogalactopyranoside (IPTG) (Gold Bio, St. Louis, MO, USA) was added to induce gene expression. Culture continued at the same settings for 4 h; then, cells were harvested via centrifugation. Cell pellets were frozen overnight at −80 °C. Pelleted cells were resuspended in 40 mL of deionized water, 2.5 mM putrescine, and 0.02% Triton X-100 and lysed via sonication. Cell lysate was clarified by centrifugation, and the supernatant was carried forward for purification via Ni-NTA chromatography (HisTrap HP, Cytivia, Wilmington, DE, USA). The running buffer contained 250 mM NaCl, 100 mM HEPES, and 5 mM imidazole at pH 7.5, and the elution buffer was identical except for increased imidazole concentration to 500 mM. Recombinant hAdoMetDC was found to elute at 190 mM imidazole. Fractions containing hAdoMetDC were further purified via dimethylaminoethyl cellulose (DEAE, BioRad, Hercules, CA, USA) ion exchange chromatography, which was equilibrated with running buffer containing 100 mM HEPES, 1 mM TCEP, 2.5 mM putrescine, and 0.1 mM EDTA, pH 7.5. The elution buffer was identical except for increased NaCl concentration to 2 M. AdoMetDC eluted at 220 mM NaCl.

### 2.2. Protein Crystallization

hAdoMetDC was concentrated to 5 mg/mL concentration in a crystallization buffer (100 mM HEPES pH 7.5, 2.5 mM putrescine, 1 mM TCEP, and 1 mM 5′-Deoxy-5′-(methylthio)adenosine (MTA)). Sitting drop vapor diffusion trays were prepared with a Formulatrix NT-8 in 2:1 protein to reservoir solution. Crystal growth occurred with conditions of 2–7% PEG 8000, 100 mM Tris-HCl, and pH 8.5, matching the previously reported crystal growth conditions [28,29,30]. For 100 K data collection, 18% glycerol containing reservoir solution was added to the crystallization drop for cryo-protection to match the previous structures. Previous reports indicate that this percentage of glycerol is vital for 100 K data collection [28,29,30]. Crystals were then cooled in the cryo-stream on Stanford Synchrotron Radiation Lightsource (SSRL) BL12-1 set to 100 K. For 273 K and 293 K data collection, crystals were not cryoprotected but were covered by RT tubes (MiTeGen, Ithaca, NY, USA) containing reservoir solution to prevent dehydration, and the cryo-stream was set to 273 K or 293 K prior to crystal mounting.

### 2.3. Data Collection and Processing

Diffraction data were collected at SSRL BL12-1 [47]. Diffraction data was collected in a single sweep from a single crystal for the 100 K and 273 K data collections. The 293 K data collection required three data collections from two crystals to be merged together in order to achieve adequate results. The automated data processing pipeline *xia2* was used to run DIALS (version 3.8) and *AIMLESS* (version 0.7.15) packages for data reduction and merging, respectively [48,49]. PDB ID 1JEN was used as the starting structure for refinement for the 100 K refinement process [28]. The new 100 K structure was used as the search model for molecular replacement in the 273 K and 293 K datasets using *Phaser* (*Phenix* version 1.20) [50]. *Phenix.refine* (*Phenix* version 1.20) was used as the automated refinement pipeline [51]. Coot was used for iterative model building [52]. The 100 K data was deposited in the Protein Data Bank with PDB ID 9P1H, and the 273 K and 293 K data were deposited as 9P7Q and 9PBB, respectively. Raw diffraction images were deposited in the SBGrid databank at data.sbgrid.org. Additional analysis was performed using Ringer and Flipper for sidechain rotamer detection [53]. RoPE was used for comparison of the torsional space with other deposited structures [54]. PASSer was used for prediction of the allosteric sites [55]. Ensemble refinement was performed with the phenix implementation using starting structures, which contained completed loops for DL1, DL2, and DL3 [56]. Structure images were created in PyMol [57]. SBGrid was used as a package manager for maintaining crystallographic software packages [58].

## 3. Results

### 3.1. Comparison of PDB ID 9P1H, the New 100 K Apo Structure, and Previously Reported Structures

**Figure 2:**
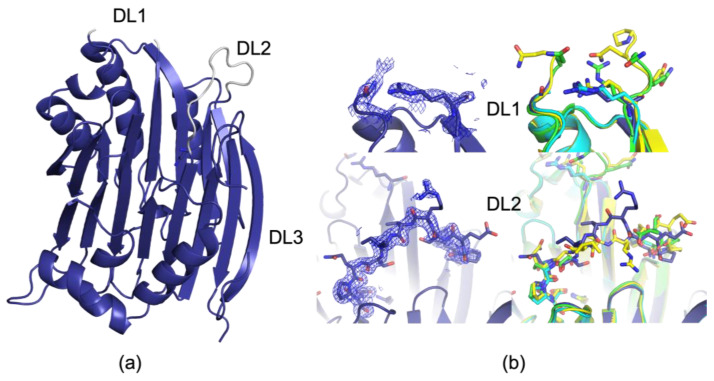
Comparison of new 100 K apo data DL1 and DL2 to previous datasets. (**a**) Overview of the AdoMetDC monomer as observed in 9P1H (indigo). DL1, DL2, and DL3 locations are shown, and DL1 and DL2 are colored in gray. (**b**) Comparison of DL1 and DL2 to previously reported structures. 2Fo-Fc maps contoured at 1.2 RMSD for 9P1H left top (DL1) and left bottom (DL2). Comparison to representative structures 1JEN (apo, 100 K, cyan), 3DZ2 (AdoMet mimic, 100 K, green), and 1I7C (MGBG, 291 K, yellow) for DL1 (right top) and DL2 (right bottom).

As a control experiment, a new 100 K crystal dataset of AdoMetDC was collected. The indexing result was P 1 2_1_ 1 with a unit cell of 73.92, 55.95, 99.17, 90, 110.78, 90, which is very similar to the original apo structure which was deposited as 1JEN [28]. The diffraction data was merged to a maximum resolution of 1.81 Å (see Table S1 for full data processing and refinement statistics of all structures reported in this manuscript). Even though crystals grew better in the presence of 1mM MTA, MTA was not detected in the electron density maps, and instead, Tris was present, as has been previously reported in the apo structure of the processing mutant, and is likely present in 1JEN as well but was not modeled due to limited resolution [24]. Upon analysis of the 100 K dataset, differences were observed between it and 1JEN, as well as other structures containing substituted AdoMet analogues or other potential inhibitors [25,30,31]. The largest deviations from this structure and previous structures are the strong densities present for disordered loop 2 (DL2), which contains residues 164–174 (Figure 2). The most comparable structure, 1JEN, does not contain coordinates for the residues between Phe164 and Gln172. Only one other structure, 1I7C, reports positions for these residues, which has methylglyoxal bis(guanylhydrazone) (MGBG) bound in the AdoMet binding site [31]. However, this loop is in a different orientation than the loop that we observe in our data (Figure 2). This indicates that there are multiple stable loop conformations which can refold preferentially depending on conditions (i.e., ligand, hydration, or temperature). Interestingly, the unique inhibitor MGBG-bound structure was collected at 291K (18 °C), making it different from the rest of the previous work in multiple ways (data collection temperature and unique ligand bound), making it challenging to pinpoint the driver of these structural shifts without additional data.

The previous structure with the most modeled residues at 100 K for DL2 is 3EP9, a structure without putrescine bound. To prepare this crystal, the putrescine was removed from the protein using perchloric acid prior to crystallization [25]. Other structures, including 1JEN, our structures, and other structures referred to as apo, contain putrescine (Figure S2). 3EP9 has a chain break from Glu166-Gln172. Interestingly, the new 100 K structure has full putrescine occupancy in both monomers, indicating that a more ordered loop does not necessarily correspond to lower putrescine occupancy, as might be surmised by a comparison of previous model structures. In the new structure, the main chain in DL2 is well defined in the 2Fo-Fc map, but the sidechains remain highly flexible and often lack interpretable density (Figure 2). Since the new 100 K form is a dimer in the asymmetric unit, two different monomers may be compared. Density is observed for both; however, the density for the loop in monomer 1 (Chains A and B) is stronger than in monomer 2 (Chains C and D).

DL1 is very similar between the new structure and previous structures, with the most glaring difference being the increased flexibility and disorder of the residues in the new data, relative to other datasets previously reported. We did not assign atomic positions to residues between Arg20 and Gly28 due to poor electron density, and this is consistent with the previously deposited apo structure, 1JEN [28]. However, the other structures, which contain inhibitors and AdoMet analogues, consistently build additional residues for DL1, often up to Pro23 and Gln27 [30]. This consistent increase in buildable positions across so many datasets of similar resolution to the new apo structure indicate that binding AdoMet or a similar molecule on the opposite side of the enzyme confers some structure to DL1. This indicates that DL1 conformation is perturbed by active site occupancy. In the absence of the new data, loss of structure in DL1 could have been assumed to be due to lower quality data in the apo dataset, but now with similar resolution apo data, that appears to not be the case.

### 3.2. The 273 K and 293 K AdoMetDC Structures Show Loop Fluctuations

Crystals grown under identical conditions to 9P1H, the new 100 K structure, were collected at 273 K and 293 K at SSRL BL12-1 in RT tubes. These crystals indexed in the C 1 2 1 space group that has been reported for many of the inhibitor and AdoMet mimic structures of AdoMetDC. This indicates that the combination of glycerol soaks and cryo-cooling are the source of the symmetry breaking in apo AdoMetDC crystals that results in the P 1 2_1_ 1 space group. More interestingly, these crystals exhibit differential behavior relative to 9P1H, our new 100 K structure, as well as the other structure collected at ambient temperatures, 1I7C, which contains the inhibitor MGBG. The 273 K and 293 K datasets are best modeled by very similar structures, indicating that there is not a large deviation in structure across this narrow temperature band.

**Figure 3:**
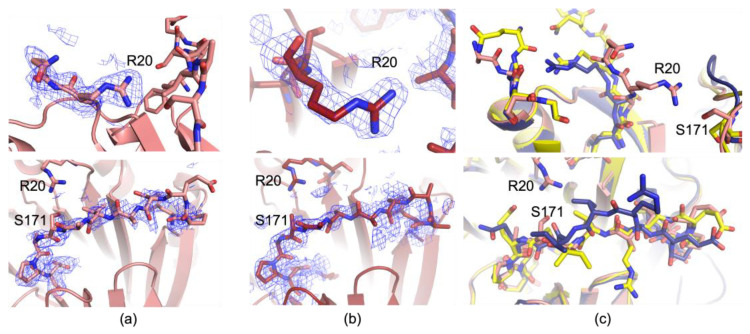
Comparison of DL1 and DL2 from 273 K and 293 K data and 1I7C. (**a**) 2Fo-Fc maps contoured at 1 RMSD for DL1 (top) and DL2 (bottom) of 273 K data. The density only enables modeling of Arg20 and Gln21 (peach, top). It enables a main chain trace through DL2, but the sidechains are poor fits to density in the middle of the loop and thus truncated. (**b**) 2Fo-Fc maps contoured at 1 RMSD for DL1 (top) and DL2 (bottom) of 293 K data. Density only enables modeling of Arg20 (red, top). The density enables a main chain trace through DL2, but the sidechains are poor fits to the density in the middle of the loop and thus truncated. (**c**) Comparison of DL1 and DL2 to previous structures. DL1 loop (273 K, peach) (top panel) is greatly altered compared to previous structures, both apo 100 K (indigo) and 1I7C, which is 291 K and inhibitor-bound (yellow). DL2’s main chain forms a structure closely resembling 1I7C (bottom panel). The 293 K data are omitted for clarity.

DL2, the loop containing residues 164–174 which is disordered in all previous structures except 1I7C, is again ordered in these datasets. However, DL2 is not in the same orientation as in 9P1H, the new 100 K dataset, but in the same position as 1I7C, the dataset collected at 291K (Figure 3). With the new 273 K and 293 K structures, there are now three total datasets, two apo and one MGBG bound, collected above the glass transition, and all three datasets contain this loop signature. It therefore appears that DL2 is ordered in ambient conditions and repacks during cryo-cooling into a more disordered state. These data imply that DL2’s structure is more dependent on data collection temperature than on the presence of an active site ligand. DL1, which contains residues 20–28, is different in both the 273 K and 293 K datasets than previously reported structures. We again do not have the requisite density to build most of the loop as in the other apo structures, 1JEN or 9P1H. However, the tails of the loop behave surprisingly and do not follow the same trend as other 100 K structures or 1I7C. In both the 273 K and 293 K apo datasets, Arg20 is rotated 180 degrees and instead of packing within DL1, it instead forms a 3.1 Å hydrogen bond with Ser171 of DL2 (Figure 3). This twist pulls the loop forward into a more compact structure, and Gln21 can also be modeled confidently. The position of Arg20 would clash with the position of Val169 of DL2 in the new 100 K structure 9P1H, with only 2.1 Å between the Arg20 nitrogen and Val169 carbon. The behavior indicates that during cryo-cooling in the apo structure, DL1 moves to a new position that is favored at cryogenic temperatures. Additionally, Arg20’s position in 1I7C being the same as in cryo structures both with and without ligands indicates that the apo structure of DL1 at 100 K is more like the inhibitor-bound state than apo ambient states.

### 3.3. Additional Analysis

To assist with placing the new structure data in context with the previous work, we used the Representation of Protein Entities (RoPE) program to compare structures in torsion angle space [54]. Even with a relatively small comparison set, patterns begin to emerge. The closest dataset to the 273 K and 293 K datasets is the 291K MGBG dataset (1I7C), even closer than our new apo 100 K structure, which came from the same protein stock and whose crystals were grown under the same conditions (Figure S3). This was especially true for the larger *α* chain, which contains DL2. This result indicates that data collection temperature is as important for the torsion angle similarity of AdoMetDC as it is for ligand occupancy. Additionally, in the cluster with the ambient data collections, we also see 3DZ3 and 3DZ5, which are two structures with covalently bound AdoMetDC mimics [30]. 3DZ3 is a Phe223 to alanine mutant with S-Adenosylmethionine methyl ester covalently bound. 3DZ5 is the wild-type enzyme with 5′-[(2-aminooxyethyl)methylamino]-5′-deoxy-8-methyladenosine adducted on the pyruvoyl group. Neither model contains atomic positions for DL1 or DL2, but it is possible that the overall torsional space is less perturbed during cryo-cooling due to the covalent linkages with the AdoMet mimics.

**Figure 4:**
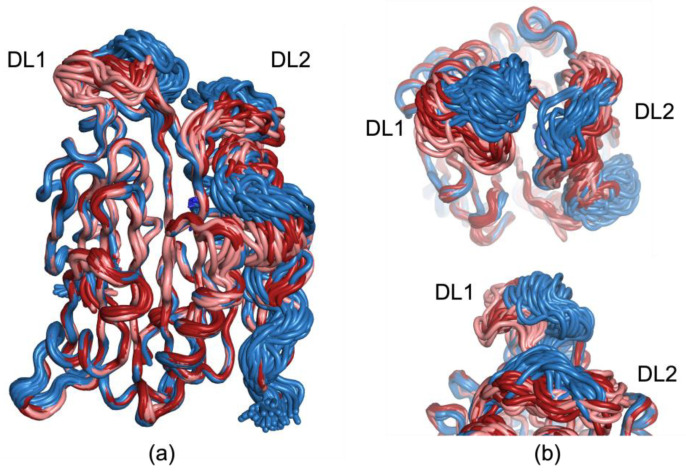
Ensemble refinements of 100 K, 273 K, and 293 K data for dynamic loop visualization. (**a**) Aligned ensemble refinements’ structure overview. The 100 K ensemble is shown in blue, the 273 K ensemble shown in peach, and the 293 K ensemble shown in red. DL1 and DL2 are labelled. (**b**) Closer inspection of DL1 and DL2. Top, forward rotation of structure in panel a shows ambient temperature data DL1 leaning forward in the cleft relative to 100 K, which has a more typical orientation for other structures in the PDB. DL2 shifts with DL1, as in 100 K, DL2 rises into a position that would clash with ambient DL1 positions. Bottom, side view of the same structures to better demonstrate how much further forward in the structure DL1 is in the ambient ensembles than at 100 K.

To better visualize the flexible loops and attempt to better understand the possible structural ensemble of the disordered residues, ensemble refinement was completed in *phenix*. The resulting r-free scores were improvements from the single deposited models for the 100 K and 273 K datasets; however, these are considered illustrative only, as clashes increase, as well as the R-work/R-free gap (Table S2). In these refinements, the 100 K structure’s DL1 residues are elongated, similarly to previously deposited structures collected at 100 K and containing ligands. Conversely, the 273 K and 293 K refinements show a more condensed DL1 loop, which folds forward more closely over the putrescine binding pocket (Figure 4). DL2 maintains the same general shape as in the deposited single models but is still quite dynamic, with its position moving towards DL1 in the 100 K model relative to the ambient temperature data collections. Disordered loop 3 (DL3), which was not able to be confidently modeled in any of our datasets, is highly mobile in the ensemble refinements. This loop appears greatly frustrated in the crystal structures, and previous structures have also struggled to model this loop.

To further investigate whether the DL1 or DL2 loops or other clefts in AdoMetDC are allosteric in nature, we submitted the 100 K structure to the Protein Allosteric Sites Server (PASSer) [55]. PASSer uses three trained machine learning models to predict allosteric sites in proteins and returns ranked scores and probabilities of pockets to be allosteric in nature. PASSer returned three possible allosteric pockets, which included the cleft between DL1 and DL2, as well as the putrescine binding site and the active site (Supplemental Figure S4). These results are also consistent with our observations of different DL1 behavior in the presence and absence of ligands bound when comparing our ambient apo data to previous structures which contained AdoMet mimics or other inhibitors.

Finally, we also used the Ringer/Flipper pipeline to analyze sidechain heterogeneity between the data collected at different temperatures (Figure S5) [33,41,53]. The sidechain analysis was inconclusive, as 273 K had increased heterogeneity relative to 100 K, while 293 K showed decreased heterogeneity. This could be due to the 293 K structure being the least structurally frustrated of the structures; however, it could also be due to differences in data quality between the crystals/datasets or some other confounding effect.

## 4. Discussion

These results suggest cryo-cooling and/or cryo-protection affects mobile loops in AdoMetDC, causing differential folding above and below the glass transition. The new 100 K structure, 9P1H, reveals a new conformation for DL2, which has not been observed before in other structures. It has moved towards the typical position of DL1 from structures containing inhibitors which have more of DL1 modeled than our structure. DL1 behaves as it does in other apo datasets in 9P1H and the placement of Arg20 is in line with the general trend for other AdoMetDC structures. These loops are therefore clearly flexible and can inhabit a wide range of conformations. In the new data reported in the present work, the structure for loop 164–174 at 100 K is a welcome change and an indication that this loop can form multiple stable orientations. It is unclear why the new 100 K structure has a more ordered DL2 loop than previous structures. The crystal conditions are very similar, with only small variations between salt and PEG conditions, as well as the same crystal space group with very similar unit cell dimensions. The crystal was also cryo-preserved, similar to previous 100 K structures in the same final concentration of glycerol. It is also probably not due to differences in crystal cooling rate or crystal age, as crystals were 1–2 weeks old, as previously reported, and many previous structures were cooled in the cold stream at the beamline or with the home source, as was the crystal in this study [28,30]. Small changes in crystal hydration may play a role, and future studies further perturbing the structural landscape using variable humidity data collection or high pressure cryo-cooling may further uncover perturbations of AdoMetDC’s structure [59,60,61,62].

Additionally, the altered conformations in 273 K and 293 K indicate that DL1 and DL2 are both affected by cryo-protection and cryo-cooling, causing them to move significantly away from the preferred structures at ambient conditions. Interestingly, the new 273 K and 293 K datasets (9P7Q and 9PBB, respectively) maintain a nearly identical mainchain trace with 1I7C, the only other ambient structure, even though it contains an inhibitor and they do not. However, Arg20 of the DL1 loop is greatly altered compared to previous structures, including 1I7C and 9P1H, the new 100 K structure. These data are highly suggestive of DL2 being temperature-sensitive, with collection temperature contributing to the altered position and order of the loop as well as the active site ligand. However, DL1 may have allosteric behavior that has been previously obscured in 100 K structures, as evidenced by the differences between ambient and cryogenic data in this work in combination with the ambient inhibitor structure from 1I7C. The new conformations observed in this work suggest that prior apo structures’ DL1 and DL2 loops shift during cryo-cooling processes, with these shifts hiding the true apo behavior of DL1 and obscuring the structure of DL2. The higher temperature structures are both more compact, with greater interplay between loops 1 and 2, including hydrogen bond formation, which occludes the interior of the protein above the putrescine binding site.

These results contribute to a growing body of literature demonstrating the importance of temperature in the structure and dynamics of proteins. Protein energy landscapes are complex and sometimes unintuitive, whereas their structure can become more ordered at higher temperatures, especially as enzymes reach their temperature of adaptation [32,35,46]. This complex behavior may partially explain the challenges involved with virtual screening and other drug design paradigms that make heavy use of PDB structures, of which the vast majority are collected at cryogenic temperatures. Such downstream efforts may be improved by protein target structural biology derived from physiological or ambient data collections, making the starting structure for the search more like the majority species in the living cell. This problem is not fully addressed by short molecular dynamics simulations used to equilibrate structures prior to docking, as the timescales of even flash cryo-cooling protein crystals allow for motions significantly slower (milliseconds) than typical all-atom molecular dynamics simulations (nanoseconds to microseconds) [63,64,65,66].

## 5. Conclusions

hAdoMetDC, an enzyme required for polyamine biosynthesis and whose activity is tightly controlled by multiple mechanisms within the cell, has long proved to be an elusive drug target to attack metabolic dysfunction in diseases including cancer. This work reports fluctuations in the structure of potentially allosteric loops, including salt bridge formation leading to a more closed conformation than previously observed. These results reinforce the usefulness of multi-temperature data collection for understanding the structure and dynamics of proteins, especially for proteins which contain flexible loop regions and other regions which may be structurally impacted by cryo-cooling and penetrating cryo-protectants. These structures also serve as alternative starting points for downstream experiments that use high-resolution structure information, such as molecular dynamics simulations and virtual screening. Loop fluctuations as detailed here would take long simulation runs to transition from the cryogenic starting points to the ambient positions, and there is no guarantee that they would ever converge on this structure without prior knowledge. Additionally, the behavior of DL1 introduces a second pocket other than the active site that potentially could be used for structure-based drug design efforts.

## Data Availability

The 100 K, 273 K, and 293 K structures were deposited in the PDB with IDs 9P1H, 9P7Q, and 9PBB, respectively, along with reflections used for refinement, scaled and unmerged data, and the map from the final round of refinement. The raw diffraction images from which those reflections were measured were deposited in the sbgrid.org databank with the following DOIs: 9P1H 100 K, https://doi.org/10.15785/SBGRID/1188; 9P7Q 273 K, https://doi.org/10.15785/SBGRID/1189; and 9PBB 293 K, https://doi.org/10.15785/SBGRID/1190.

## Supplementary Materials

The following supporting information can be downloaded at: https://www.mdpi.com/article/10.3390/biom15091274/s1, Table S1: Diffraction data processing and refinement statistics for PDB depositions; Figure S1: Location of binding pockets in relation to DL1 and DL2; Figure S2: Putrescine pocket of 100 K, 273 K, and 293 K structures; Figure S3: RoPE analysis of AdoMetDC alpha chain; Table S2: Ensemble refinement statistics from phenix.ensemble_refinement; Figure S4: Protein Allosteric Sites Server (PASSer) results; Figure S5: Ringer/Flipper peak gain/loss analysis of AdoMetDC.

## Abbreviations

The following abbreviations are used in this manuscript:

ODC	Ornithine decarboxylase
AdoMetDC	S-adenosylmethionine decarboxylase
SPDS	Spermidine synthase
SPS	Spermine synthase
AdoMet	S-adenosylmethionine
dcAdoMet	Decarboxylated S-adenosylmethionine
hAdoMetDC	Human S-adenosylmethionine decarboxylase
DL1	Disordered loop 1
DL2	Disordered loop 2
DL3	Disordered loop 3
*AMD1*	Human AdoMetDC gene
IPTG	Isopropyl *β*-D-1-thiogalactopyranoside
MTA	5′-Deoxy-5′-(methylthio)adenosine
MGBG	Methylglyoxal bis(guanylhydrazone)
RoPE	Representation of Protein Entities
PASSer	Protein Allosteric Sites Server
