# Supplementary material for: Multi-Temperature Crystallography of S-Adenosylmethionine Decarboxylase Observes Dynamic Loop Motions
Source: Biomolecules. 2025 Sep 3;15(9):1274. doi: 10.3390/biom15091274 (PMC12467396; doi:10.3390/biom15091274)
Supplement: Supplementary file 1 [file biomolecules-15-01274-s001.zip › biomolecules-3761175-supplementary.pdf]

Supplemental Figures for "Multi-temperature Crystallography of S-adenosylmethionine Decarboxylase Observes Dynamic Loop Motions"

Supplemental Table S1. Diffraction data processing and refinement statistics for PDB depositions

	**9P1H 100 K**	**9P7Q 273 K**	**9PBB 293 K**
Wavelength	0.97946	0.9795	0.9795
Resolution range	43.48 - 1.807 (1.86 - 1.81)	34.86 - 2.21 (2.34 - 2.21)	37.50 - 2.17 (2.20 - 2.16)
Space group	P 1 2_1 1	C 1 2 1	C 1 2 1
Unit cell	73.85 55.95 99.25 90 110.88 90	96.98 45.02 72.06 90 105.11 90	97.42 45.88 72.25 90 104.97 90
Total reflections	225636 (17299)	46664 (7540)	116951 (1958)
Unique reflections	67064 (5042)	14760 (2215)	16461 (706)
Multiplicity	3.4 (3.4)	3.1 (3.2)	7.1 (2.8)
Completeness (%)	96.9 (98.0)	96.00 (92.69)	98.7 (85.4)
Mean I/sigma(I)	9.4 (1.2)	4.5 (2.0)	5.1 (1.50)
Wilson B-factor	26.81	11.01	16.14
R-merge	0.074 (1.671)	0.286 (1.578)	0.306 (0.932)
R-meas	0.088 (1.983)	0.347 (1.909)	0.331 (1.159)
R-pim	0.047 (1.054)	0.192 (1.057)	0.123 (0.71)
CC1/2	0.998 (0.301)	0.942 (0.34)	0.977 (0.285)
Reflections used in refinement	66958 (2526)	14760 (2215)	16253 (2391)
Reflections used for R-free	3370 (119)	791 (130)	815 (127)
R-work	0.1806 (0.3194)	0.2197 (0.2784)	0.2080 (0.2808)
R-free	0.1993 (0.3063)	0.2588 (0.3100)	0.2228 (0.2898)
Number of non-hydrogen atoms	5382	2623	2628
macromolecules	4964	2492	2476
ligands	56	20	19
solvent	362	111	133
Protein residues	616	308	307
RMS(bonds)	0.008	0.01	0.015
RMS(angles)	0.73	1.08	1.04
Ramachandran favored (%)	96.5	95.00	96.66
Ramachandran allowed (%)	3.5	4.33	3.01
Ramachandran outliers (%)	0	0.67	0.33
Rotamer outliers (%)	0.18	1.79	0.72
Clashscore	4.25	3.63	4.68
Average B-factor	35.86	35.41	29.20
macromolecules	35.46	35.52	28.97
ligands	41.16	32.94	33.19
solvent	40.56	33.32	32.90

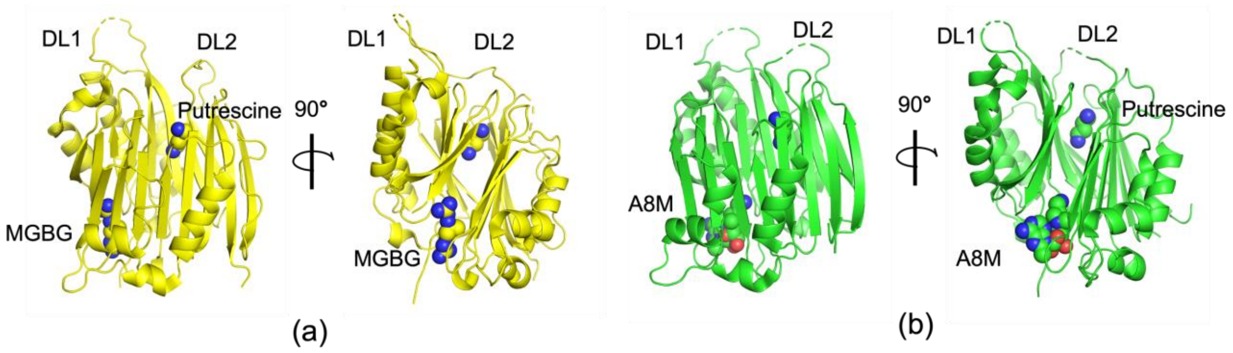

Supplemental Figure S1. Location of binding pockets in relation to DL1 and DL2. (a) 1I7C structure (yellow) containing inhibitor MGBG bound in the active site and putrescine bound in its binding site. DL1 and DL2 are labeled showing their distal location relative to the active site. (b) 3DZ2 structure (green) with AdoMet mimic 5'-[(3-aminopropyl)methylamino]-5'deoxy-8-methyladenosine (labelled A8M) bound in the active site and putrescine in its binding pocket.

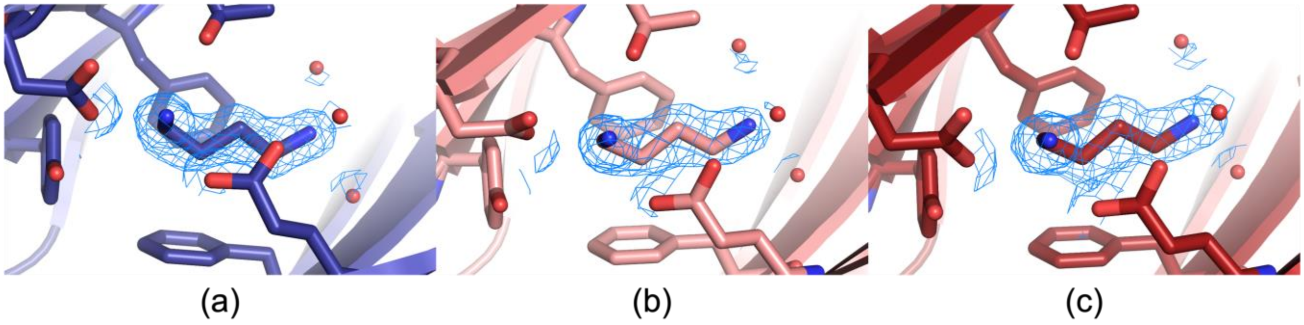

Supplemental Figure S2. Putrescine pocket of 100 K, 273 K, and 293 K structures. (a) 100 K structure (9P1H) shown in indigo with putrescine density shown at 1.2 RMSD. (b) 273 K structure (9P7Q) shown in peach with putrescine density shown at 1.2 RMSD. (c) 293 K structure (9PBB) shown in red with putrescine density shown at 1.2 RMSD.

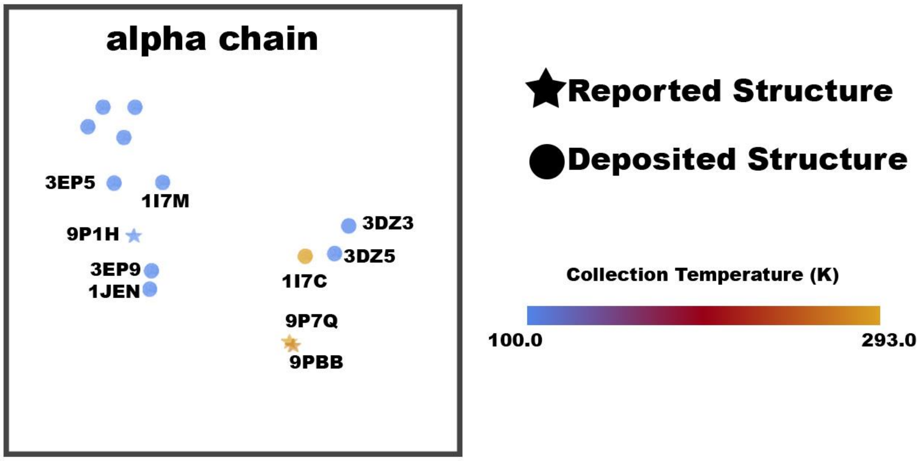

Supplemental Figure S3. RoPE analysis of AdoMetDC alpha chain. PDB IDs of notable apo (3EP5, 3EP9, and 1JEN) and ligand bound (3DZ3, 3DZ5, and 1I7C) are shown. There is clear differential clustering with ambient collections associating with the covalently bound AdoMet derivatives as opposed to the other 100K structures.

Supplemental Table S2. Ensemble refinement statistics from phenix.ensemble_refinement

	100K ensemble refinement	273K ensemble refinement	293K ensemble refinement
R-Work	0.1590	0.1703	0.1731
R-Free	0.2052	0.2351	0.2256
Number of models	67	29	23

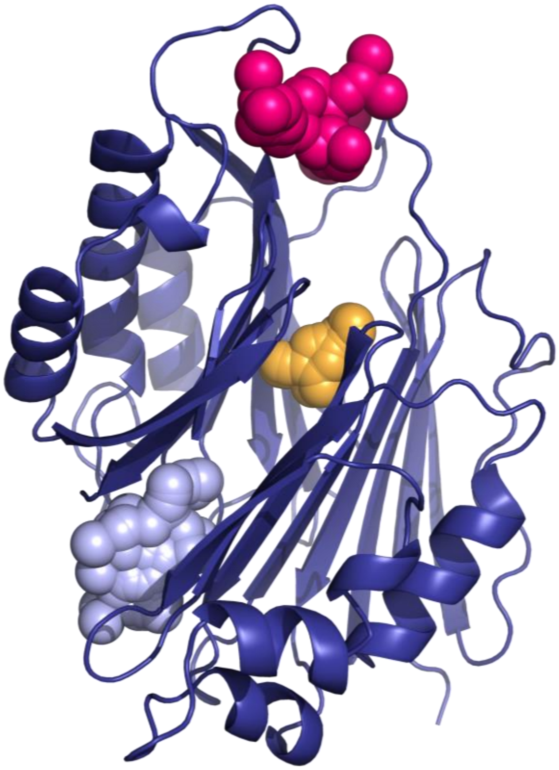

Supplemental Figure S4. Protein Allosteric Sites Server (PASSer) results. Used an all-loops version of the 100K SAMDC structure as the starting model for analysis (dark blue). Top 3 sites found were the active site (pale blue spheres, 83% probability score), the putrescine binding site (orange, 60% probability score), and ordered loop area (magenta, 40% probability score).

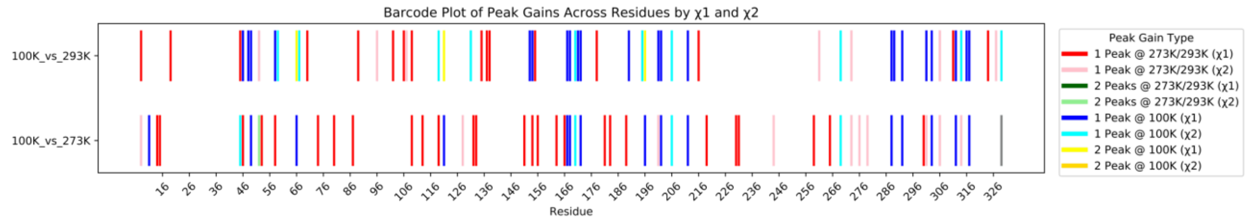

Supplemental Figure S5. Ringer/Flipper peak gain/loss analysis of AdoMetDC. In comparison to the structure of AdoMetDC at 100K, more peak gains were observed at 273K, whereas the 293K structure showed more peak losses.
